# Development of a mixed feed strategy for a recombinant *Pichia pastoris* strain producing with a de-repression promoter

**DOI:** 10.1186/s12934-015-0292-7

**Published:** 2015-07-10

**Authors:** Simona Capone, Jernej Horvat, Christoph Herwig, Oliver Spadiut

**Affiliations:** Institute of Chemical Engineering, Research Area Biochemical Engineering, Vienna University of Technology, Gumpendorfer Strasse 1a, 1060 Vienna, Austria

**Keywords:** *Pichia pastoris*, Mutated AOX1 promoter, De-repression, Phospholipase C, Glycerol, Sorbitol, Mixed feed, Productivity

## Abstract

**Background:**

Recombinant protein production in the yeast *Pichia pastoris* is usually based on the alcohol oxidase promoters pAOX1 and pAOX2, which are regulated by methanol and strongly repressed by other C-sources, like glycerol and glucose. However, the use of methanol brings several disadvantages, which is why current trends in bioprocess development with *P. pastoris* are focussing on minimizing the required amount of methanol or even avoid its employment. In this respect novel promoter systems which do not rely on methanol have been investigated and promoter variants were designed to fine-tune gene expression. Amongst these novel promoter systems, mutated AOX promoters, which are regulated by available carbon source concentration (so-called de-repressed promoters), are currently raising attention. However, the main disadvantage of such a production system is that expression and growth usually cannot happen concomitantly resulting in low space–time-yields.

**Results:**

Here we show the development of a mixed-feed strategy for an industrial recombinant *P. pastoris* de-repression strain aiming at increased productivity and maximum space–time-yield. By doing dynamic experiments we determined a ratio between the specific substrate uptake rates of glycerol and sorbitol allowing a more than 2-fold increased productivity compared to the conventional single substrate de-repression strategy.

**Conclusion:**

Based on our results we recommend adjusting q_s glycerol_ = 0.04 g g^−1^ h^−1^ and q_s sorbitol_ = 0.055 g g^−1^ h^−1^ to obtain highest productivity with a *P. pastoris* de-repression strain. Our methodological approach of designing mixed-feed strategies based on physiological strain characterization using dynamic experiments proved to be beneficial.

**Electronic supplementary material:**

The online version of this article (doi:10.1186/s12934-015-0292-7) contains supplementary material, which is available to authorized users.

## Background

The methylotrophic yeast *Pichia pastoris* is widely used for recombinant protein production in industrial biotechnology. Recombinant protein production in this yeast is usually based on the transcriptional activity of the alcohol oxidase promoters pAOX1 and pAOX2, which are regulated by methanol and strongly repressed by other C-sources, like glycerol and glucose [1]. However, the use of methanol brings several disadvantages as methanol metabolism causes heat production and increased need of oxygen, on-line methanol monitoring and especially feedback control is difficult and methanol accumulation leads to the production of toxic compounds [2]. Consequently, current trends in bioprocess development with *P. pastoris* are focussing on minimizing the required amount of methanol.

Bioprocesses employing *P. pastoris* can on the one hand be improved by developing mixed feed strategies, where a primary C-source (e.g. glycerol) is used for biomass growth in non-repressing concentrations, whereas methanol is used for induction [2–4]. In this respect, positive effects of mixed-feed strategies on productivity have been demonstrated for both *P. pastoris* Mut^S^ and Mut^+^ strains [5]. To analyze the benefit of a mixed-feed strategy on productivity in more detail, recent studies are aiming at a better understanding of its impact on cell physiology by performing metabolomics and metabolic flux analysis [2, 6]. In a recent study, we were able to show considerable improvement by determining the specific substrate uptake rates (q_s_) and employing dynamic experiments to examine physiological conditions allowing high productivity in a methanol-glycerol mixed feed environment [7]. However, even in these mixed feed systems methanol is still required for induction making it less attractive for industrial large-scale production processes.

Besides bioprocess engineering approaches, also strain engineering can be applied to minimize or even avoid the use of methanol as inducer. Novel promoter systems which do not rely on methanol have been investigated and promoter variants were designed to fine-tune gene expression [8–11]. Amongst these novel promoter systems, mutated AOX promoters, which are regulated by carbon source depletion (so-called de-repressed promoters), are currently raising attention [8, 12]. These promoter systems are especially interesting for bioreactor cultivations, where substrate concentrations can be tightly controlled at levels allowing full promoter de-repression. However, the main disadvantage of such a production system is that expression and growth usually cannot happen concomitantly. Thus, a typical bioprocess with these systems usually comprises three steps: (1) batch, (2) repressed fed-batch at a high growth rate, and (3) de-repressed fed-batch at limited carbon source concentration for production. Thus, space–time-yields (STY) with these systems are typically rather low.

In the present study we physiologically characterized an industrial *P. pastoris* strain harbouring the recombinant product phospholipase C (PLC) from *Bacillus cereus* under the control of a mutated AOX1 promoter, which gets de-repressed at limiting concentrations of glycerol. Due to high structural and catalytic similarity of PLC from *B. cereus* with mammalian PLCs, it is currently used as a good model system and is thus a highly important subject in medical research [13]. Since this enzyme is hard to produce, the current price for an enzyme preparation from *B. cereus* lies at 320 Euros for 5 Units, which corresponds to only 5 μg of protein (P5542-5UN; Sigma Aldrich).

Based on physiological parameters, we cultivated the recombinant *P. pastoris* strain under optimized conditions following the typical feeding regime for such de-repressed strains comprising of the three phases (1) batch, (2) repressed fed-batch at high q_s glycerol_ and (3) de-repressed fed-batch at low q_s glycerol_. However, our main goal was the development of a mixed feed strategy for this strain aiming at increased productivity and a higher STY. Thus, we also propose a novel methodology to develop a mixed-feed strategy for industrial recombinant *P. pastoris* de-repression strains using dynamic experiments.

## Methods

### Microorganism

A *P. pastoris* CBS7435 Mut^S^ strain carrying the recombinant gene for phospholipase C from *Bacillus cereus* (PLC; EC 3.1.4.3) under the control of a mutated AOX1 promoter, conferring high expression upon de-repressing conditions (*i.e.* at limiting glycerol concentrations), was kindly provided by VTU Technology GmbH (Grambach, Austria). This strain will be referred to as “recombinant *P. pastoris* de-repression strain” in this article.

### Culture media

Precultures were done in yeast nitrogen base medium (YNB; 0.1 M potassium phosphate buffer pH 6.0, 3.4 g L^−1^ YNB w/o amino acids and ammonia sulfate, 10 g L^−1^ (NH_4_)_2_SO_4_, 400 mg L^−1^ biotin, 20 g L^−1^ glucose). Zeocine was added to a concentration of 100 μg L^−1^.

Batch and fed-batch cultivations were performed in 2-fold concentrated basal salt medium (BSM; 21.6 mL L^−1^ 85% phosphoric acid, 0.36 g L^−1^ CaSO_4_∙2H_2_O, 27.24 g L^−1^ K_2_SO_4_, 4.48 g L^−1^ MgSO_4_∙7H_2_O, 8.26 g L^−1^ KOH, 0.3 mL L^−1^ Antifoam Struktol J650, 4.35 mL L^−1^ PTM1, NH_4_OH as N-source). Trace element solution (PTM1) was made of 6.0 g L^−1^ CuSO_4_·5H_2_O, 0.08 g L^−1^ NaI, 3.0 g L^−1^ MnSO_4_·H_2_O, 0.2 g L^−1^ Na_2_MoO_4_·2H_2_O, 0.02 g L^−1^ H_3_BO_3_, 0.5 g L^−1^ CoCl_2_, 20.0 g L^−1^ ZnCl_2_, 65.0 g L^−1^ FeSO_4_·7H_2_O, 0.2 g L^−1^ biotin, 5 mL L^−1^ H_2_SO_4_. The concentration of the base NH_4_OH was determined by titration with 0.25 M potassium hydrogen phthalate.

### Bioreactor cultivations

#### Preculture

Frozen stocks (−80°C) were cultivated in 100 mL YNB-Zeocine in 1,000 mL shake flasks at 30°C and 230 rpm for 24 h. Then, the preculture was transferred aseptically to the culture vessel. The inoculum volume was 10% (v/v) of the final starting volume.

#### Batch and fed-batch cultivations

Batch and fed-batch cultivations were carried out in a 5 L working volume glass bioreactor (Infors, Switzerland). Dissolved oxygen (dO_2_) was measured with a sterilizable fluorescence dissolved oxygen electrode (Visiferm DO425, Hamilton, Germany). The pH was measured with a sterilizable electrode (Easyferm™, Hamilton, Switzerland) and maintained constant at pH 5.0 with a PID controller using NH_4_OH (2–3 M). Base consumption was determined gravimetrically. Cultivation temperature was set to 30°C and agitation was fixed to 1,000 rpm. The culture was aerated with 2.0 vvm dried air to keep dissolved oxygen level above 30%. In case of dO_2_ limitation pure oxygen was added. Off-gas was measured by an infrared cell for CO_2_ and a zirconium dioxide sensor for O_2_ concentration (DasGip, Germany). Temperature, pH, dO_2_, agitation as well as CO_2_ and O_2_ in the off-gas were measured online and logged in a process information management system (PIMS; Lucullus, Biospectra, Switzerland).

Before fed-batch experiments, a single dynamic batch cultivation with substrate pulses was performed, as we described previously [14–16], to determine the 2nd C-source for the development of a mixed feed strategy. After the complete consumption of glucose at a concentration of 40 g L^−1^ in the batch, the C-sources glucose, sorbitol, mannose, fructose, maltose, glycerol and lactic acid were sequentially pulsed twice to the culture each in a final concentration of 45 mM. For each pulse, at least two samples were taken for offline sample analysis to calculate specific rates and yields.

Fed-batch experiments were conducted as follows: after a batch on a C-source at a final concentration of 40 g L^−1^, either a dynamic or an exponential fed-batch was performed, where the feeding rate based on q_s_ was constantly adjusted according to the total amount of biomass in the bioreactor and controlled by the PIMS. Real-time measurement of total biomass was done by a soft-sensor tool as we described previously [17]. Off-line measurements of biomass were done every 2 h to correct for potential soft sensor errors. Dynamic feeding was controlled by a built-in online calculator [18] according to Eq. 1:

1 $${\text{F}} = \frac{{{\text{q}}_{\text{s}} {\text{ theoretical }} \times {\text{X }} \times \rho \;{\text{feed }} \times {\text{reactor weight }}}}{{{\text{S }} \times \rho {\text{broth }}}}$$

q_s_ theoretical = q_s_ set point (g g^−1^ h^−1^); X = biomass estimated by soft sensor (g L^−1^); ρ feed = density of feed (g L^−1^); reactor weight (g); S = feed concentration (g L^−1^); ρ broth = density of culture broth (g L^−1^).

The different fed-batch experiments, the respective feeding rates as well as the respective goals of each experiment are summarized in Table 1.

**Table 1:** Overview of dynamic fed-batch experiments

Experiment	Substrate	Feeding strategy based on q_s_ (g g^−1^ h^−1^)	Goals
FB1	Glycerol	Batch on glycerol–step-wise decrease of q_s glycerol_: 0.338–0.063–0.054–0.029–0.014–0.005 g g^−1^ h^−1^	Determination of strain physiological parameters Characterization of recombinant expression profile
FB2	Glycerol	Batch on glycerol–repression phase on glycerol (q_s_ = 0.29 g g^−1^ h^−1^)–de-repression phase on glycerol (q_s_ = 0.035 g g^−1^ h^−1^)	Mimic optimized industrial process comprising of three phases
FB3	Sorbitol	Batch on sorbitol–step-wise increase of q_s sorbitol_: 0.033–0.060–0.127–0.176–0.197 g g^−1^ h^−1^	Determination of strain physiological parameters Characterization of recombinant expression profile
		Glycerol pulse at a final concentration of 10 g L^−1^ at highest q_s sorbitol_	q_s_ in presence of both substrates
FB4	Mixed feed	Batch on glycerol–repression phase on glycerol (q_s_ = 0.33 g g^−1^ h^−1^)–de-repression phase on glycerol (q_s_ = 0.054 g g^−1^ h^−1^)–mixed-feed: glycerol (q_s_ = 0.054 g g^−1^ h^−1^) and sorbitol (q_s_ = 0.070 g g^−1^ h^−1^)–mixed-feed: glycerol (q_s_ = 0.026 g g^−1^ h^−1^) and sorbitol (q_s_ = 0.015 g g^−1^ h^−1^)	Analyze physiology and productivity in mixed feed environment
FB5	Mixed feed	batch on glycerol– repression phase on glycerol (q_s_ = 0.33 g^−1^ h^−1^)–mixed-feed: glycerol (q_s_ = 0.040 g^−1^ h^−1^) and sorbitol (q_s_ = 0.055 g g^−1^ h^−1^)	Verify increased STY in mixed feed environment
FB6	Mixed feed	batch on glycerol–repression phase on glycerol (q_s_ = 0.23 g g^−1^ h^−1^)–mixed-feed: glycerol (q_s_ = 0.026 g g^−1^ h^−1^) and sorbitol (q_s_ = 0.027 g g^−1^ h^−1^)–mixed-feed: glycerol (q_s_ = 0.026 g g^−1^ h^−1^) and sorbitol (q_s_ = 0.061 g g^−1^ h^−1^)	Verify increased STY in mixed feed environment Identification of q_s__glycerol_/q_s sorbitol_ ratio allowing highest productivity and STY

### Offline sample analysis

#### Analysis of growth and expression parameters

Dry cell weight (DCW) was determined by centrifugation of 5 mL culture broth (5,000 rpm, 4°C, 10 min) in a laboratory centrifuge (Sigma 4K15, rotor 11,156), washing the pellet with 5 mL deionized water and subsequent drying at 105°C to a constant weight in an oven. Optical density at 600 nm (OD_600_) was measured in a photometer (U-1100 Hitachi, Japan). A linear correlation between DCW and OD_600_ was experimentally determined (Eq. 2).

2 $${\text{DCW }}\left( {{\text{g}}\;{\text{L}}^{ - 1} } \right) \, = \, \left( {0. 50 6\times {\text{OD}}_{ 600} + 0.000 6} \right) \times {\text{dilution factor}}$$

Since the regression coefficient R^2^ was 0.996, Eq. 2 was used for regular q_s_ adjustments of the feed based on OD_600_ measurements.

#### Substrates and metabolites

Concentrations of carbon sources and metabolites were determined in cell free cultivation broth using HPLC (Agilent Technologies, USA), equipped with a Supelcoguard column, a Supelcogel C-610 H ion exchange column (Sigma-Aldrich, USA) and a refractive index detector (Agilent Technologies, USA). The mobile phase was 0.1% H_3_PO_4_ with a constant flow rate of 0.5 mL min^−1^ and the system was run isocratically at 30°C. All measurements were executed in duplicates.

#### PLC activity assay

Recombinant PLC activity in cell-free cultivation broth was determined with a colorimetric method based on the hydrolysis of *p*-nitrophenylphosphorylcholine (*p*-NPPC) [19]. Quantification was done with a photometer (U-1100 Hitachi, Japan). The standard curve was prepared with commercial PLC from *B. cereus* (Sigma-Aldrich, P6621-250UN) dissolved in HEPES buffer (50 mM, pH 7.0) and *p*-NPPC (Melford, 21064-69-7) substrate solution (100 mM in 250 mM HEPES buffer, 0.1 mM ZnCl_2_, pH 7.0, 30% sorbitol). 540 µL of cell-free cultivation broth were mixed with 60 µL of *p*-NPPC substrate solution. The mixture was incubated at 37°C for 60 min [20] and enzymatic activity was followed at 410 nm [21]. Total extracellular protein content was determined by the Bradford Reagent (Sigma-Aldrich, B6919) at 595 nm.

## Results and discussion

### Dynamic batch cultivation with substrate pulses

**Figure 1:**
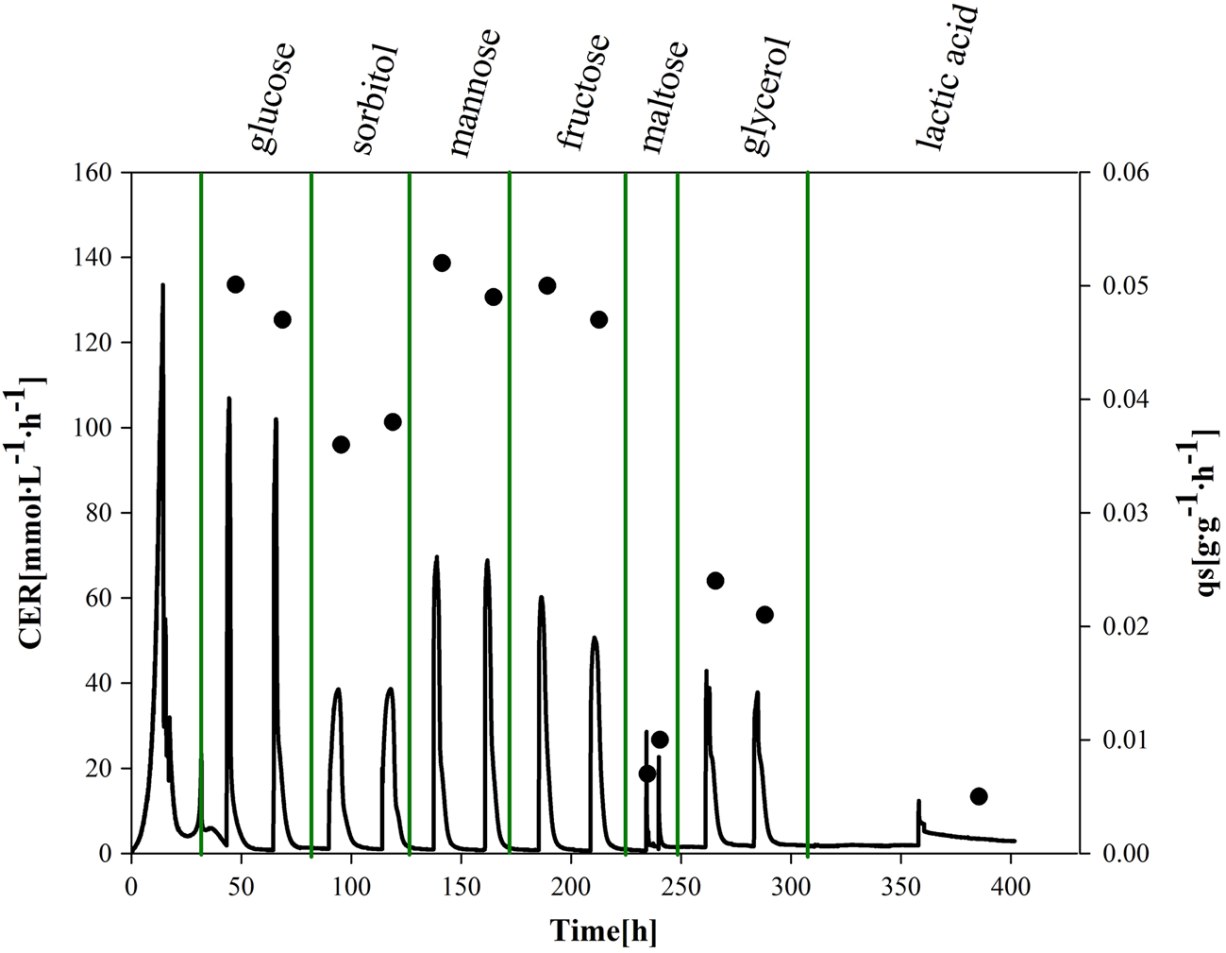
Dynamic batch cultivation with substrate pulses. Glucose, sorbitol, mannose, fructose, maltose, glycerol and lactic acid were sequentially pulsed after a batch on glucose. The CER signal (*continuous black line*) was used to follow metabolic activity. The specific substrate uptake rate (q_s_, *black dot*) was determined for the different substrates.

To identify a second C-source for developing the mixed-feed strategy for the recombinant *P. pastoris* de-repression strain, we performed a dynamic batch cultivation with substrate pulses. After a batch on glucose, the C-sources glucose, sorbitol, mannose, fructose, maltose, glycerol and lactic acid were sequentially pulsed twice to the culture each in a final concentration of 45 mM. In Figure 1 the carbon dioxide evolution rate (CER), depicting metabolic activity, and the calculated specific substrate uptake rates (q_s_) are shown.

Based on offline analysis, specific rates and yields were calculated. We also analyzed productivity, formation of metabolites as well as economic aspects. As shown in Table 2, all substrates were taken up; however, maltose and lactic acid were only metabolized at low rates. Metabolism of glucose and mannose led to the formation of ethanol, which could be problematic in mixed feed experiments. We analyzed the volumetric productivity (r_p_) for each substrate pulse and found promoter de-repression in each phase. However, fructose and lactic acid gave lower r_p_ compared to the other substrates. Closing C-balances confirmed data validity (Table 2). Taken together, based on physiology, productivity and economic aspects we chose sorbitol as 2nd C-source for the development of a mixed feed strategy.

**Table 2:** Summary of strain specific parameters, metabolites and STY during dynamic batch experiment with substrate pulses as well as economic aspects

C-source	q_s_ (g g^−1^ h^−1^)	${\text{Y}}_{{{\text{CO}}_{2} /{\text{S}}}}$ (mol Cmol^−1^)	Y_X/S_ (Cmol Cmol^−1^)	C-balance	Metabolites	r_p_ (U L^−1^ h^−1^) per pulse	Price (€ kg^−1^)
Glucose (batch)	0.059	0.53	0.498	1.03	Acetate (3.0 g L^−1^) Ethanol (0.6 g L^−1^)	6.2	
Glucose	0.048	0.93	0.004	0.94	Ethanol (0.3 g L^−1^)	4.2	40
Sorbitol	0.037	0.94	0.002	0.94	nd	4.8	20
Mannose	0.051	0.93	0.008	0.94	ethanol (1.0 g L^−1^)	4.7	2,000
Fructose	0.048	0.90	0.036	0.94	nd	2.2	62
Maltose	0.006	0.93	0.014	0.94	nd	5.4	252
Glycerol	0.023	1.04	0.011	1.05	nd	4.3	26
Lactic acid	0.005	1.04	0.000	1.04	nd	0.5	24

*q*_*s*_ specific substrate uptake rate, ${\text{Y}}_{{{\text{CO}}_{2} /{\text{S}}}}$ *and Y*_*X/S*_ yields of CO_2_ and biomass, *C-balance* sum of ${\text{Y}}_{{{\text{CO}}_{2} /{\text{S}}}}$and Y_X/S_ which should ideally give 1.0, *r*_*p*_ volumetric productivity per pulse and *nd* none determined.

### Fed-batch cultivations

#### Dynamic fed-batch on glycerol as sole carbon-source (FB1)

In order to physiologically characterize the recombinant *P. pastoris* de-repression strain and identify the glycerol concentration allowing for full promoter de-repression, we performed FB1 where we stepwise adapted the feeding rate to correspond to lower q_s glycerol_ (Table 1; Additional file 1: Figure S1).

As expected, decreasing q_s_._glycerol_ resulted in a decreased specific growth rate (µ) and a decreased Y_X/S_ (Table 3). In particular, by adjusting the feeding rate to correspond to a q_s glycerol_ between 0.029 g g^−1^ h^−1^ and 0.054 g g^−1^ h^−1^ we could nicely determine the maintenance level of this yeast strain (Additional file 2: Figure S2). At q_s glycerol_ lower than 0.05 g g^−1^ h^−1^, the cells use the C-source for maintenance metabolism and not for growth, which is shown in increasing ${\text{Y}}_{{{\text{CO}}_{2} /{\text{S}}}}$ values. Only at q_s glycerol_ higher than 0.05 g g^−1^ h^−1^, the cells can efficiently produce biomass and product.

**Table 3:** Strain characteristic parameters during the dynamic fed-batch on glycerol (FB1)

q_s glycerol_ (g g^−1^ h^−1^)	µ (h^−1^)	${\text{Y}}_{{{\text{CO}}_{2} /{\text{S}}}}$ (mol Cmol^−1^)	Y_X/S_ (Cmol Cmol^−1^)	C-balance	r_p_ (U L^−1^ h^−1^)	q_p_ (U g^−1^ h^−1^)
0.338	0.199	0.29	0.70	0.99	2.45	0.03
0.063	0.026	0.52	0.47	0.99	5.38	0.06
0.054	0.024	0.43	0.53	0.96	54.8	0.57
0.029	0.008	0.61	0.34	0.95	13.8	0.14
0.014	0.000	0.91	0.03	0.94	13.5	0.13
0.005	0.000	1.01	0.01	1.02	6.08	0.06

When we analyzed r_p_ and q_p_ during the single q_s glycerol_ steps, we determined apparent full promoter de-repression at a q_s glycerol_ = 0.054 g g^−1^ h^−1^ (Table 3). Interestingly, by further lowering q_s glycerol_, also productivity decreased. Apparently, maximum productivity for the recombinant *P. pastoris* de-repression strain is directly linked to an optimum q_s glycerol_. As soon as the cells come close to their maintenance level, r_p_ and q_p_ decrease dramatically, leaving only a narrow operating window for the design of an efficient de-repression fed-batch (Additional file 3: Figure S3, Additional file 4: Figure S4).

#### Production fed-batch on glycerol as sole carbon-source (FB2)

Based on data from FB1, we performed FB2 to mimic an optimized industrial process comprising of the three phases (1) batch on glycerol, (2) repressed fed-batch at high q_s glycerol_ and (3) de-repressed fed-batch at low q_s glycerol_ (Table 1). After the batch, we cultivated the cells at a repressing q_s glycerol_ = 0.29 g g^−1^ h^−1^ to a biomass concentration of 60 g L^−1^. Then we adjusted the feeding rate to correspond to a de-repressing q_s glycerol_ = 0.035 g g^−1^ h^−1^ and continued the cultivation for another 24 h. The final biomass concentration was 65 g L^−1^. In Table 4 the physiological strain characteristic parameters are summarized. In the de-repressed phase we determined r_p_ = 23.6 U L^−1^ h^−1^ and q_p_ = 0.36 U g^−1^ h^−1^.

**Table 4:** Strain characteristic parameters during the production fed-batch on glycerol (FB2)

q_s glycerol_ (g g^−1^ h^−1^)	µ (h^−1^)	${\text{Y}}_{{{\text{CO}}_{2} /{\text{S}}}}$ (mol Cmol^−1^)	Y_X/S_ (Cmol Cmol^−1^)	C-balance	r_p_ (U L^−1^ h^−1^)	q_p_ (U g^−1^ h^−1^)
0.29	0.104	0.40	0.58	0.98	0	0
0.035	0.012	0.73	0.30	1.03	23.6	0.36

#### Dynamic fed-batch on sorbitol as sole carbon-source (FB3)

To characterize the recombinant *P. pastoris* de-repression strain on sorbitol, we performed a batch on sorbitol which was followed by a fed-batch where we stepwise increased q_s sorbitol_ (Table 1; Additional file 5: Figure S5).

We observed an extremely long lag phase during the batch at a concentration of 40 g L^−1^ sorbitol (Additional file 5: Figure S5). We speculate that an osmotic shock caused by the high sorbitol concentration could have caused this long lag phase [22, 23]. However, when we analyzed the different q_s sorbitol_ steps, we observed a nice correlation between q_s sorbitol_, μ and Y_X/S_ (Table 5) and thus were again able to determine the maintenance level of the strain between q_s sorbitol_ = 0.060 and 0.127 g g^−1^ h^−1^ (Additional file 6: Figure S6).

**Table 5:** Strain characteristic parameters during the dynamic fed-batch on sorbitol (FB3)

q_s sorbitol_ (g g^−1^ h^−1^)	µ (h^−1^)	${\text{Y}}_{{{\text{CO}}_{2} /{\text{S}}}}$ (mol Cmol^−1^)	Y_X/S_ (Cmol Cmol^−1^)	C-balance	r_p_ (U L^−1^ h^−1^)	q_p_ (U g^−1^ h^−1^)
0.033	0.005	0.76	0.16	0.92	0.63	0.028
0.060	0.018	0.57	0.36	0.93	1.37	0.032
0.127	0.059	0.47	0.55	1.02	4.74	0.051
0.176	0.084	0.45	0.56	1.01	10.4	0.170
0.197	0.088	0.48	0.53	1.01	33.0	0.951

When we raised q_s sorbitol_ >0.197 g g^−1^ h^−1^, we observed sorbitol accumulation. Interestingly, productivity increased concomitantly with q_s sorbitol_ and the substrate did not repress the promoter at any concentration (Table 5; Additional file 7: Figure S7, Additional file 8: Figure S8). At the end of cultivation we determined a biomass concentration of around 40 g L^−1^.

**Figure 2:**
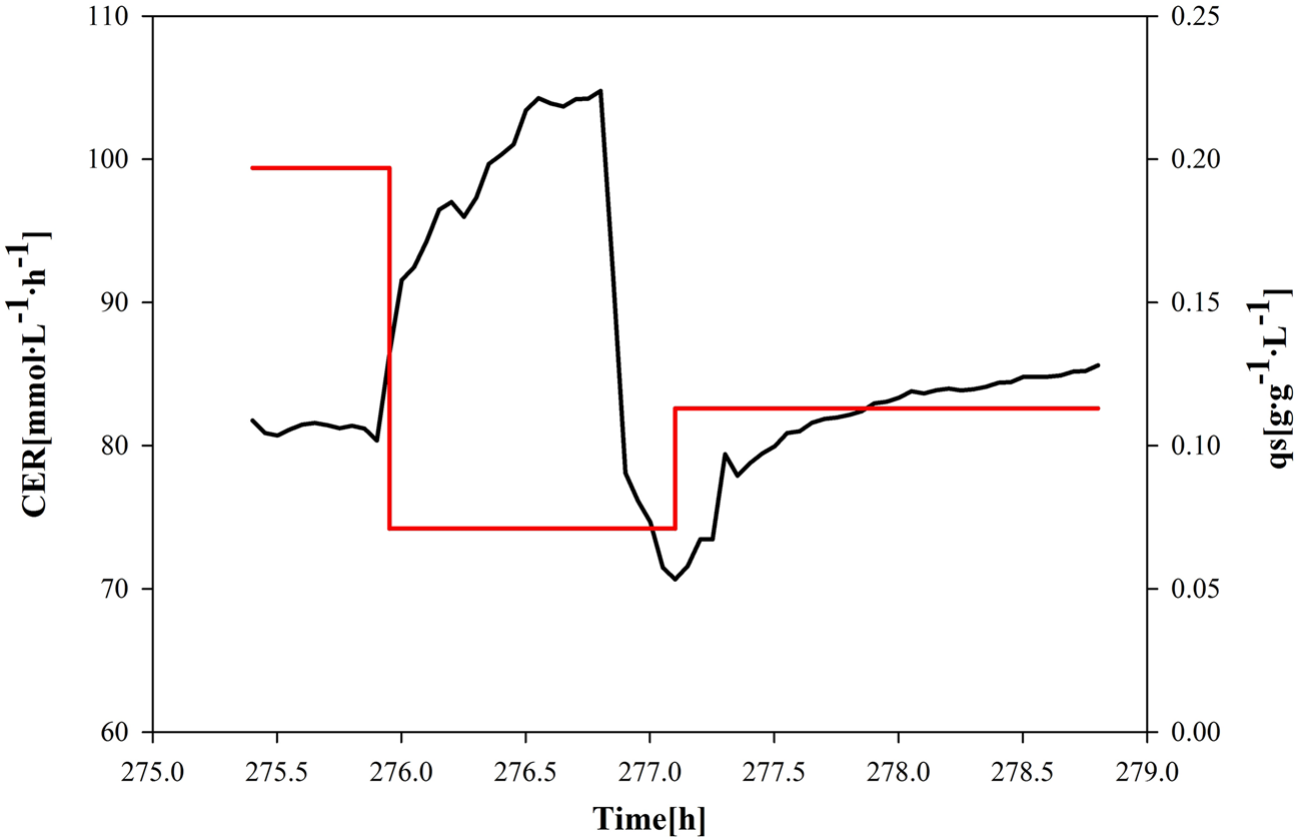
Glycerol pulse during sorbitol fed-batch. Carbon dioxide evolution rate (CER, *continuous black line*) and specific sorbitol uptake rate (q_s sorbitol_, *continuous red line*). After glycerol pulse a sudden increase in the CER signal and a concomitant decrease in q_s__sorbitol_ were observed.

To analyze if sorbitol and glycerol can be taken up concomitantly and identify the respective q_s_ values, we pulsed glycerol at a final concentration of 10 g L^−1^ to the culture while sorbitol was fed at a constant q_s sorbitol_ = 0.197 g g^−1^ h^−1^. The cells immediately reacted to glycerol, as indicated by a sudden increase in the CER (Figure 2).

Sampling before and after the glycerol pulse and offline analysis allowed the determination of physiological parameters. As shown in Table 6, glycerol was specifically taken up at a rather high rate of 0.193 g g^−1^ h^−1^, whereas q_s sorbitol_ decreased from 0.197 to 0.071 g g^−1^ h^−1^. Although the recombinant *P. pastoris* de-repression strain preferred glycerol as substrate, both C-sources were taken up concomitantly. This was a crucial observation, as the concomitant uptake was an essential requirement for the development of a mixed feed strategy. As expected, we did not determine an increase of the total amount of PLC in the cultivation broth after pulsing glycerol in a repressing concentration. However, we even determined a lower total amount of active PLC in the cultivation broth after the pulse, indicating PLC to be a very unstable product, which constantly degraded and/or lost activity in the bioreactor.

**Table 6:** Strain characteristic parameters during a glycerol pulse in sorbitol fed-batch FB3 (glycerol was pulsed at a final concentration of 10 g L^−1^ to the culture while sorbitol was fed at a constant q_s sorbitol_ = 0.197 g g^−1^ h^−1^)

q_s glycerol_ (g g^−1^ h^−1^)	q_s sorbitol_ (g g^−1^ h^−1^)	µ (h^−1^)	${\text{Y}}_{{{\text{CO}}_{2} /{\text{S}}}}$ (mol Cmol^−1^)	Y_X/S_ (Cmol Cmol^−1^)	C-balance	r_p_ (U L^−1^ h^−1^)	q_p_ (U g^−1^ h^−1^)
0.193	0.071	0.186	0.26	0.71	0.97	0^*^	0^*^

* We even measured less active PLC after the glycerol pulse than before, indicating product instability.

#### Dynamic mixed feed fed-batch (FB4)

In order to verify q_s_ for both glycerol and sorbitol, we performed a dynamic mixed feed fed-batch. After a fed-batch phase on glycerol at a repressing concentration of q_s glycerol_ = 0.33 g g^−1^ h^−1^ to a biomass concentration of around 40 g L^−1^, we de-repressed the promoter at a q_s glycerol_ = 0.054 g g^−1^ h^−1^ for 24 h to get a biomass concentration of around 60 g L^−1^. Then we added the 2nd C-source sorbitol and performed two different mixed feed phases: first we concomitantly fed glycerol at q_s glycerol_ = 0.054 g g^−1^ h^−1^ and sorbitol at q_s sorbitol_ = 0.070 g g^−1^ h^−1^ for 8 h giving a biomass concentration of 78 g L^−1^, before we adapted the feeding rates to correspond to a q_s glycerol_ = 0.026 g g^−1^ h^−1^ and q_s sorbitol_ = 0.015 g g^−1^ h^−1^ for another 52 h resulting in a biomass concentration of around 66 g L^−1^. The results of this dynamic experiment are summarized in Table 7.

**Table 7:** Strain characteristic parameters during dynamic mixed feed fed-batch FB4

q_s glycerol_ (g g^−1^ h^−1^)	q_s sorbitol_ (g g^−1^ h^−1^)	µ (h^−1^)	${\text{Y}}_{{{\text{CO}}_{2} /{\text{S}}}}$ (mol Cmol^−1^)	Y_X/S_ (Cmol Cmol^−1^)	C-balance	r_p_ (U L^−1^ h^−1^)	q_p_ (U g^−1^ h^−1^)
0.33	–	0.174	0.38	0.63	1.01	0	0
0.054	–	0.018	0.63	0.42	1.05	5.24	0.080
0.054	0.070	0.082	0.44	0.57	1.01	13.25	0.143
0.026	0.015	0.011	0.80	0.20	1.00	5.57	0.099

As shown in Table 7, the concomitant presence of sorbitol boosted μ more than 4-fold from 0.018 to 0.082 h^−1^. We could also follow the positive effect of sorbitol on cell growth by shifts in both yields. When we reduced q_s_ for both substrates in the later phase of the mixed feed fed-batch, also μ dramatically decreased. In terms of productivity, we obtained a 2-fold increase in the first mixed feed phase compared to the glycerol de-repression phase. When we decreased both q_s_ values, also productivities decreased. Interestingly, for the de-repression phase at q_s glycerol_ = 0.054 g g^−1^ h^−1^ we only obtained r_p_ = 5.24 U L^−1^ h^−1^ and q_p_ = 0.08 U g^−1^ h^−1^ which was 4-fold lower compared to the results obtained in FB2 at q_s glycerol_ = 0.035 g g^−1^ h^−1^. Based on our observations in FB1, we actually expected an even higher productivity at q_s glycerol_ = 0.054 g g^−1^ h^−1^. We currently have no explanation for this mismatch, however we speculate that a different batch of p-NPPC substrate might have caused this aberration. Thus, for the following experiments we always used the same batch of substrate. However, for FB4 a direct comparison between the different phases was still possible and legitimate, showing a beneficial effect of a mixed feed environment on productivity compared to a single substrate de-repression strategy.

#### Production mixed feed fed-batch (FB5)

In order to verify the higher productivity observed for a mixed feed environment compared to a de-repression strategy on glycerol as sole C-source, we performed FB5. First we performed a de-repressed fed-batch phase at a q_s glycerol_ = 0.33 g g^−1^ h^−1^ to a biomass concentration of 65 g L^−1^, before we started the mixed feed where we kept q_s_ values for both substrates constant (Table 8).

**Table 8:** Strain characteristic parameters during production mixed feed fed-batch FB5

q_s glycerol_ (g g^−1^ h^−1^)	q_s sorbitol_ (g g^−1^ h^−1^)	µ (h^−1^)	${\text{Y}}_{{{\text{CO}}_{2} /{\text{S}}}}$ (mol Cmol^−1^)	Y_X/S_ (Cmol Cmol^−1^)	C-balance	r_p_ (U L^−1^ h^−1^)	q_p_ (U g^−1^ h^−1^)
0.33	–	0.189	0.32	0.69	1.01	0	0
0.040	0.055	0.062	0.41	0.63	1.04	64.7	0.86

Compared to the production fed-batch on glycerol as sole carbon-source (FB2), where we determined μ = 0.012 h^−1^, r_p_ = 23.6 U L^−1^ h^−1^ and q_p_ = 0.36 U g^−1^ h^−1^ at a q_s glycerol_ = 0.035 g g^−1^ h^−1^ (Table 3), we increased μ 5-fold to μ = 0.062 h^−1^, r_p_ 2.8-fold to r_p_ = 65.7 U L^−1^ h^−1^ and q_p_ 2.4-fold to q_p_ = 0.86 U g^−1^ h^−1^ at a comparable q_s glycerol_ in a mixed feed environment with concomitant uptake of sorbitol. This clearly shows the beneficial effect of the mixed feed system on productivity and STY compared to the single substrate strategy.

#### Production mixed feed fed-batch (FB6)

**Figure 3:**
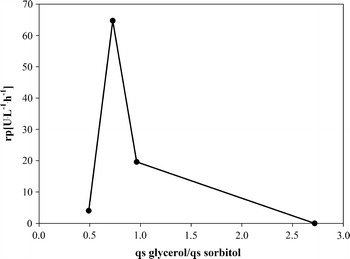
Mechanistic plot depicting the correlation between the ratio of $\frac{{{\text{q}}_{\text{s glycerol}} }}{{{\text{q}}_{\text{s sorbitol}} }}$ and the volumetric productivity (r_p_).

**Figure 4:**
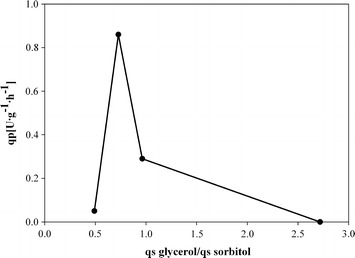
Mechanistic plot depicting the correlation between the ratio of $\frac{{{\text{q}}_{\text{s glycerol}} }}{{{\text{q}}_{\text{s sorbitol}} }}$ and the specific productivity (q_p_).

To determine a potential optimal ratio between q_s glycerol_ and q_s sorbitol_, we performed FB6, where, after a repression phase on glycerol to a biomass concentration of 60 g L^−1^, we tested 2 different mixed feed environments (Table 9). The productivity values determined in the first mixed feed phase were comparable to the values determined in the previous fed-batch experiments. Interestingly, by keeping q_s glycerol_ constant and increasing q_s sorbitol_ around 2-fold we increased μ, but significantly decreased the productivity (Table 9). This was rather surprising, since we expected to further increase productivity by increasing q_s sorbitol_ and thus μ. However, the results suggest, that the boost in productivity does not result from a higher μ, but rather from a certain ratio of q_s glycerol_ to q_s sorbitol_. Thus, we plotted the ratio $\frac{{{\text{q}}_{\text{s glycerol}} }}{{{\text{q}}_{\text{s sorbitol}} }}$ of FB3, FB5 and FB6 against productivity values (Figures 3, 4). Due to the different substrate batches for activity measurements and thus possibly non-comparable productivity values, we did not consider FB4 for this mechanistic plot. As shown in Figures 3 and 4, a ratio $\frac{{{\text{q}}_{\text{s glycerol}} }}{{{\text{q}}_{\text{s sorbitol}} }}$ of around 0.7 is most beneficial for productivity.

**Table 9:** Strain characteristic parameters during production mixed feed fed-batch FB6

q_s glycerol_ (g g^−1^ h^−1^)	q_s sorbitol_ (g g^−1^ h^−1^)	µ (h^−1^)	${\text{Y}}_{{{\text{CO}}_{2} /{\text{S}}}}$ (mol Cmol^−1^)	Y_X/S_ (Cmol Cmol^−1^)	C-balance	r_p_ (U L^−1^ h^−1^)	q_p_ (U g^−1^ h^−1^)
0.23	–	0.127	0.35	0.67	1.02	0	0
0.026	0.027	0.022	0.52	0.50	1.02	19.6	0.29
0.026	0.061	0.053	0.30	0.72	1.02	4.03	0.05

## Conclusions

In this study we physiologically characterized a recombinant *P. pastoris* strain, where the gene coding for the recombinant product phospholipase C (PLC) was under the control of a mutated AOX1 promoter, which gets de-repressed at limiting concentrations of glycerol. Based on physiological data we developed a mixed feed strategy for this novel de-repression strain and compared productivity data to the commonly used single substrate strategy. Our findings can be summarized as:

- A dynamic batch with substrate pulses revealed sorbitol as second C-source for the development of a mixed feed strategy.
- The specific substrate uptake rate for glycerol allowing full promoter de-repression was determined with q_s glycerol_ = 0.054 g g^−1^ h^−1^. However, maximum productivity could only be achieved in a rather small operating window of q_s glycerol_, which underlines the importance of precise and robust process control.
- Sorbitol did not repress the mutated AOX1 de-repression promoter. The maximum specific substrate uptake rate of this strain for sorbitol was determined with q_s sorbitol_ = 0.197 g g^−1^ h^−1^, where also highest productivity was reached.
- An easy-to-perform pulse experiment showed that the yeast strain was able to take up both glycerol and sorbitol concomitantly and revealed maximum q_s_ values for both substrates.
- The highest productivity was reached at a ratio of q_s glycerol_ to q_s sorbitol_ of 0.7. In this study, we were able to boost the productivity more than 2-fold in the mixed feed environment compared to the commonly used single substrate strategy, where we fed glycerol in de-repressing conditions.

Summarizing, we have employed a methodological approach based on dynamic experiments to establish a mixed-feed strategy for a recombinant *P. pastoris* de-repression strain comprising three phases (1) batch, (2) repressed fed-batch at high q_s glycerol_ and (3) de-repressed mixed feed fed-batch at a ratio q_s glycerol_ to q_s sorbitol_ of 0.7. Based on our results we recommend adjusting q_s glycerol_ = 0.04 g g^−1^ h^−1^ and q_s sorbitol_ = 0.055 g g^−1^ h^−1^ to obtain highest productivity. Our methodological approach of designing mixed-feed strategies based on physiological strain characterization using dynamic experiments proved to be beneficial.

## Additional files

Additional file 1: 
**Figure S1.** Dynamic fed-batch on glycerol as sole carbon source (FB1). The carbon dioxide evolution rate signal (CER, continuous black line) was used to follow metabolic activity. The specific glycerol uptake rate (q_s glycerol_) is depicted as continuous red line. Additional file 2:
**Figure S2.** Carbon dioxide yield (${\text{Y}}_{{{\text{CO}}_{2} /{\text{S}}}}$, black dots) and biomass yield (Y_X/S,_ white squares) at different specific glycerol uptake rates (q_s glycerol_). Additional file 3: **Figure S3.** Volumetric productivity (r_p_) at different specific glycerol uptake rates (q_s glycerol_). Additional file 4: 
**Figure S4.** Specific productivity (q_p_) at different specific glycerol uptake rates (q_s glycerol_). Additional file 5:
** Figure S5.** Dynamic fed-batch on sorbitol as sole carbon source (FB3). The carbon dioxide evolution rate signal (CER, continuous black line) was used to follow metabolic activity. The specific sorbitol uptake rate (q_s sorbitol_) is depicted as continuous red line. Additional file 6:
** Figure S6.** Carbon dioxide yield (${\text{Y}}_{{{\text{CO}}_{2} /{\text{S}}}}$, black dots) and biomass yield (Y_X/S_, white squares) at different specific sorbitol uptake rates (q_s sorbitol_). Additional file 7:
**Figure S7.** Volumetric productivity (r_p_) at different specific sorbitol uptake rates (q_s sorbitol_). Additional file 8: 
**Figure S8.** Specific productivity (q_p_) at different specific sorbitol uptake rates (q_s sorbitol_).
