# Supplementary figures and images for: Development of a mixed feed strategy for a recombinant Pichia pastoris strain producing with a de-repression promoter
Source: Microb Cell Fact. 2015 Jul 10;14:101. doi: 10.1186/s12934-015-0292-7 (PMC4561368; doi:10.1186/s12934-015-0292-7)

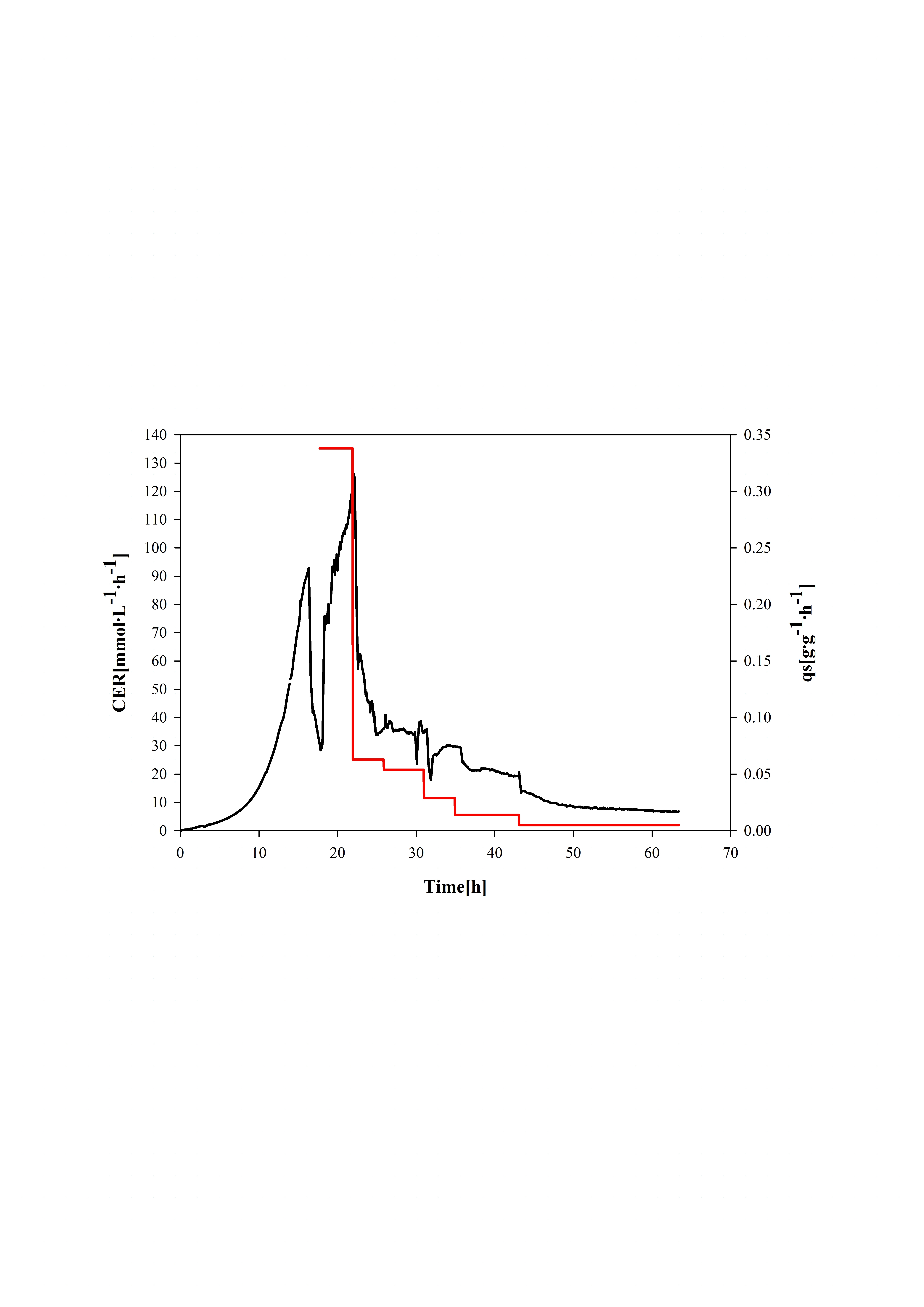

Supplement: Additional file 1: — Figure S1. Dynamic fed-batch on glycerol as sole carbon source (FB1). The carbon dioxide evolution rate signal (CER, continuous black line) was used to follow metabolic activity. The specific glycerol uptake rate (qs glycerol) is depicted as continuous red line. [file 12934_2015_292_MOESM1_ESM.jpg]

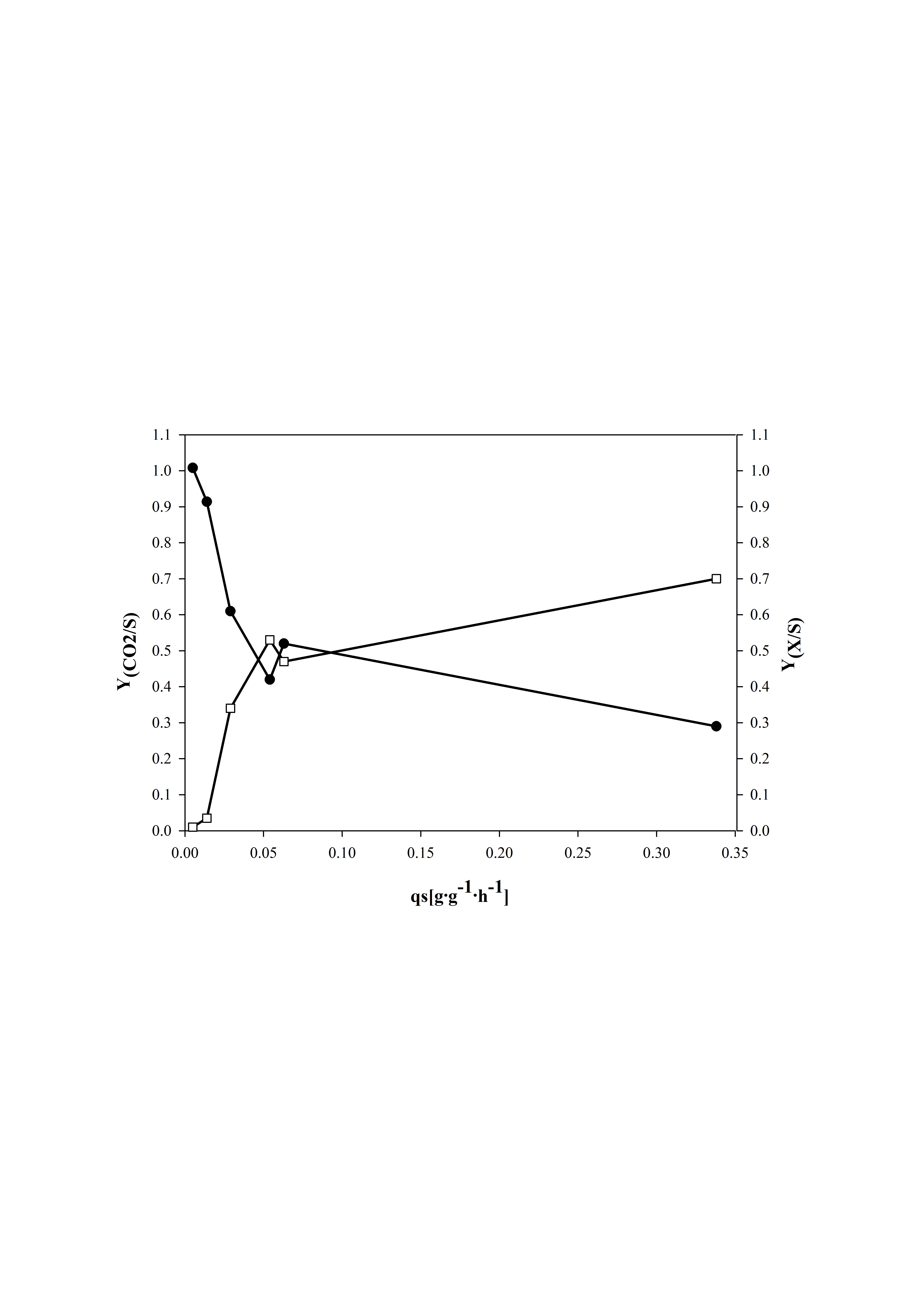

Supplement: Additional file 2: — Figure S2. Carbon dioxide yield (${\text{Y}}_{{{\text{CO}}_{2} /{\text{S}}}}$YCO2/S, black dots) and biomass yield (YX/S, white squares) at different specific glycerol uptake rates (qs glycerol). [file 12934_2015_292_MOESM2_ESM.jpg]

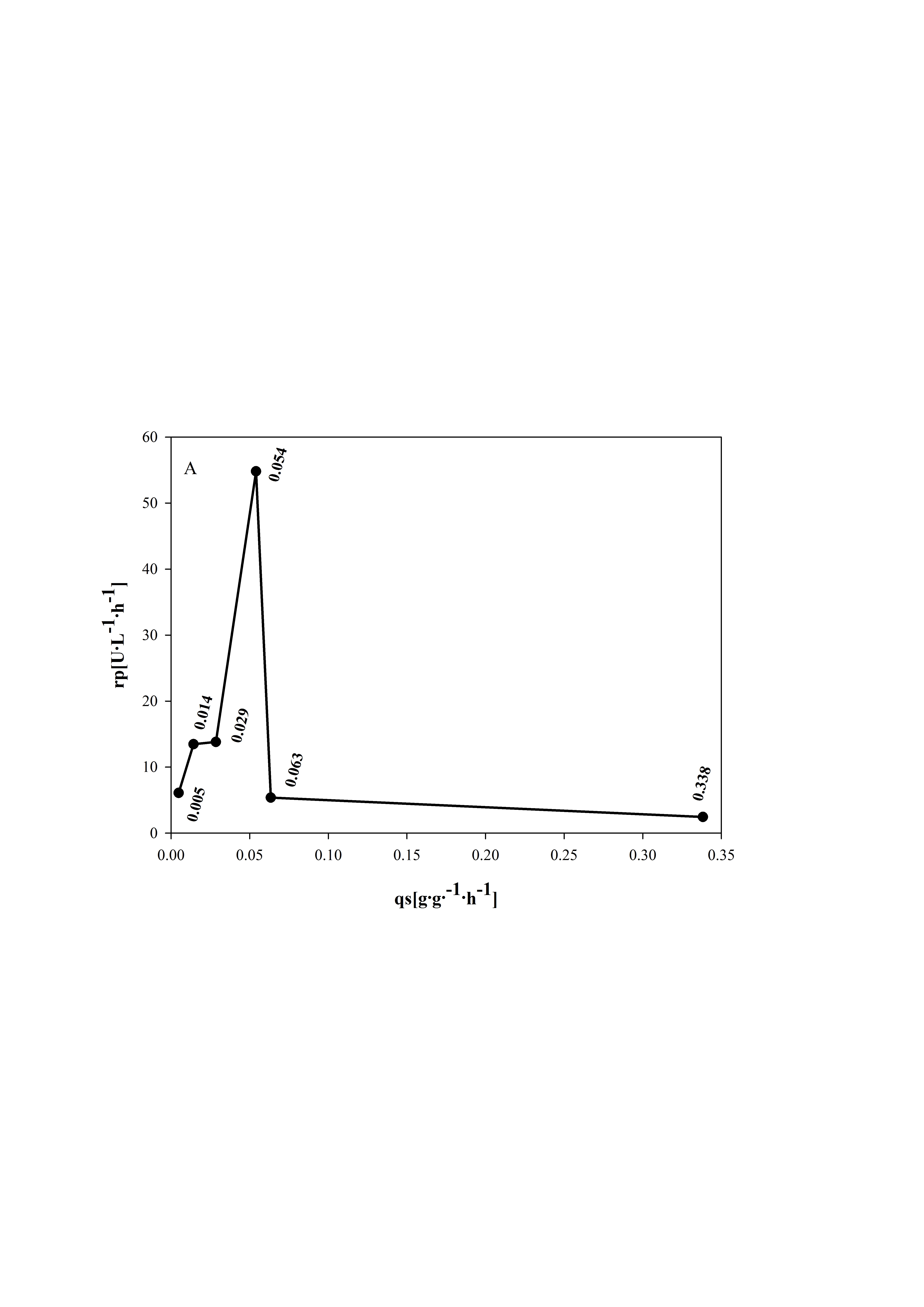

Supplement: Additional file 3: — Figure S3. Volumetric productivity (rp) at different specific glycerol uptake rates (qs glycerol). [file 12934_2015_292_MOESM3_ESM.jpg]

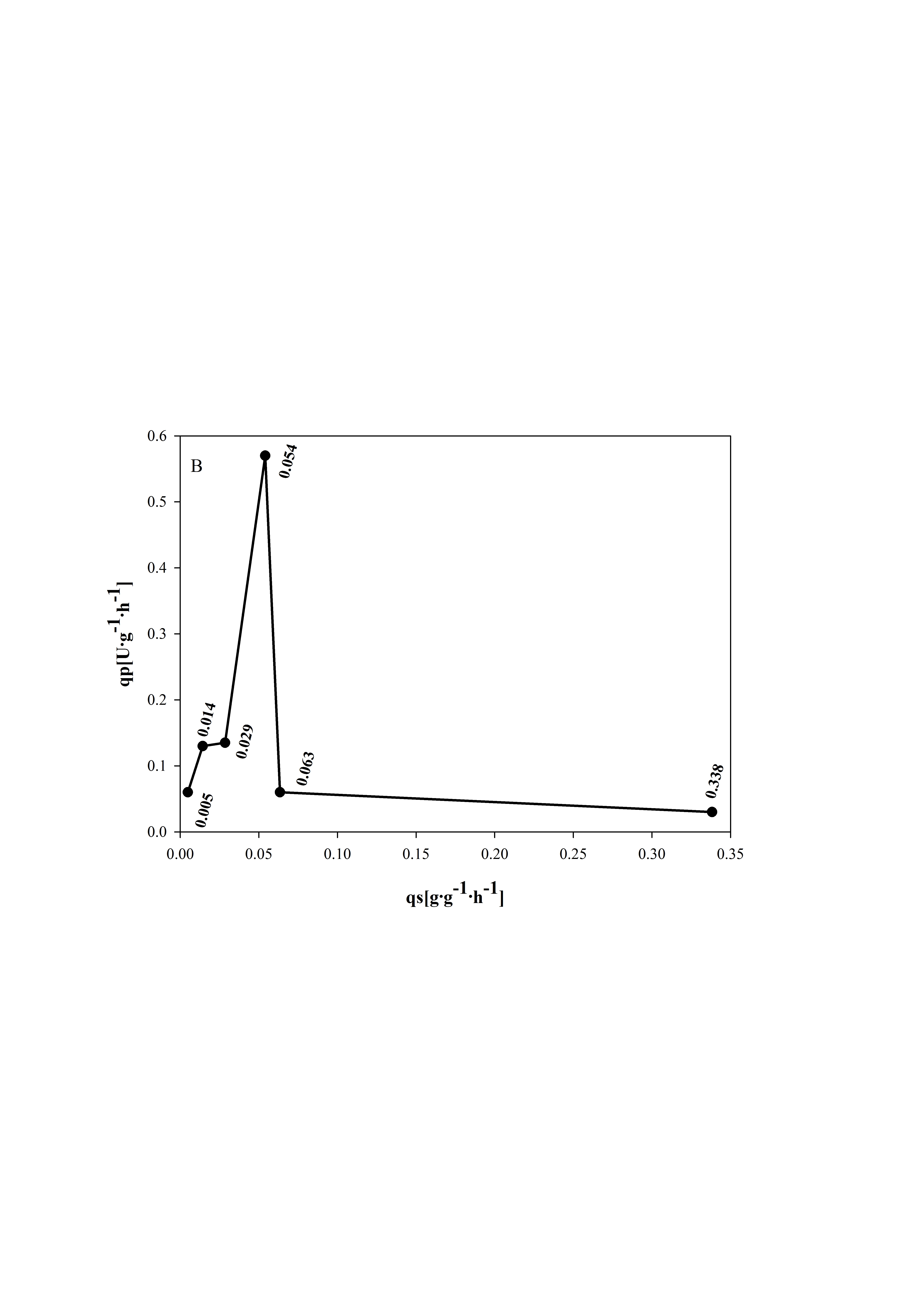

Supplement: Additional file 4: — Figure S4. Specific productivity (qp) at different specific glycerol uptake rates (qs glycerol). [file 12934_2015_292_MOESM4_ESM.jpg]

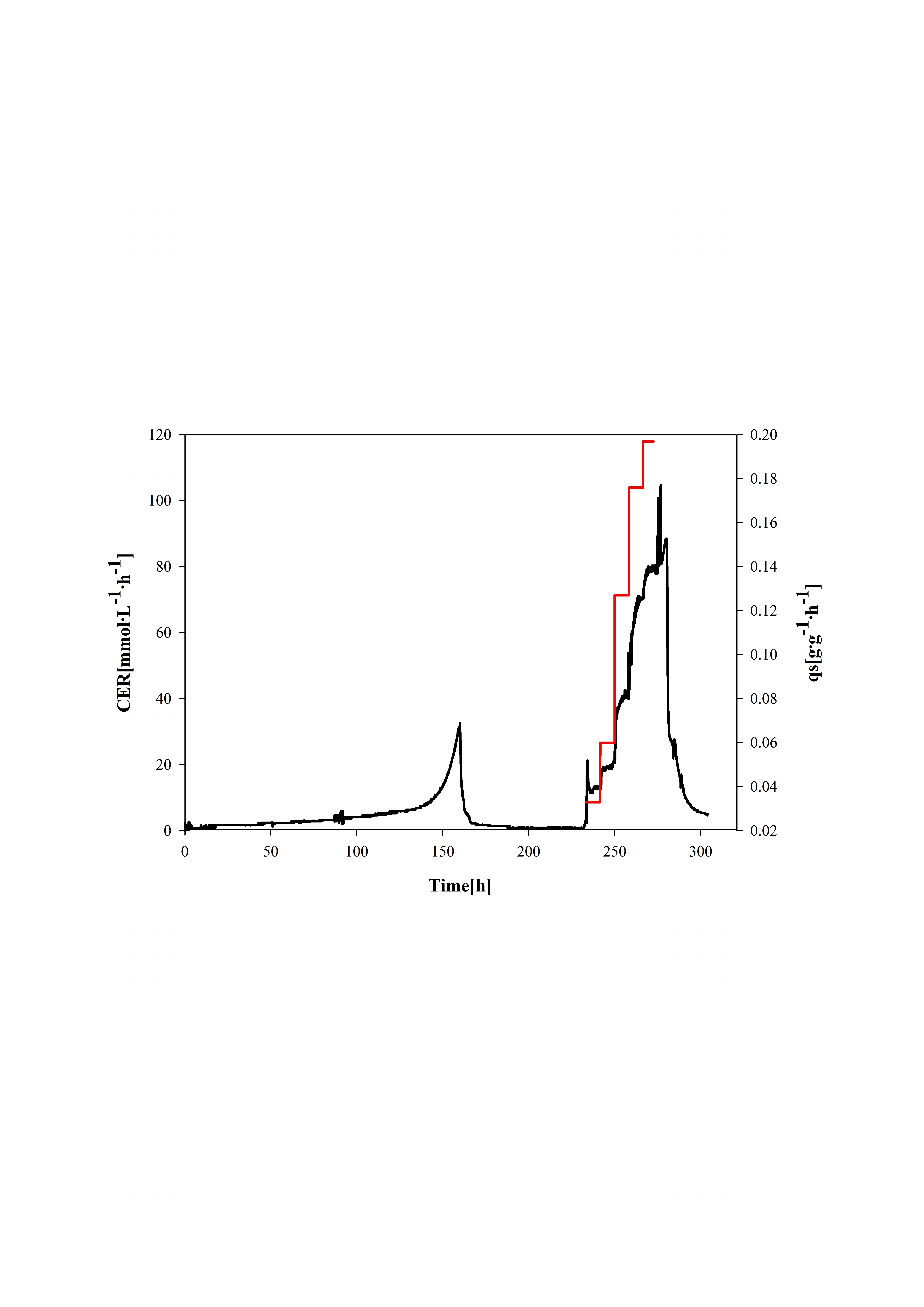

Supplement: Additional file 5: — Figure S5. Dynamic fed-batch on sorbitol as sole carbon source (FB3). The carbon dioxide evolution rate signal (CER, continuous black line) was used to follow metabolic activity. The specific sorbitol uptake rate (qs sorbitol) is depicted as continuous red line. [file 12934_2015_292_MOESM5_ESM.jpg]

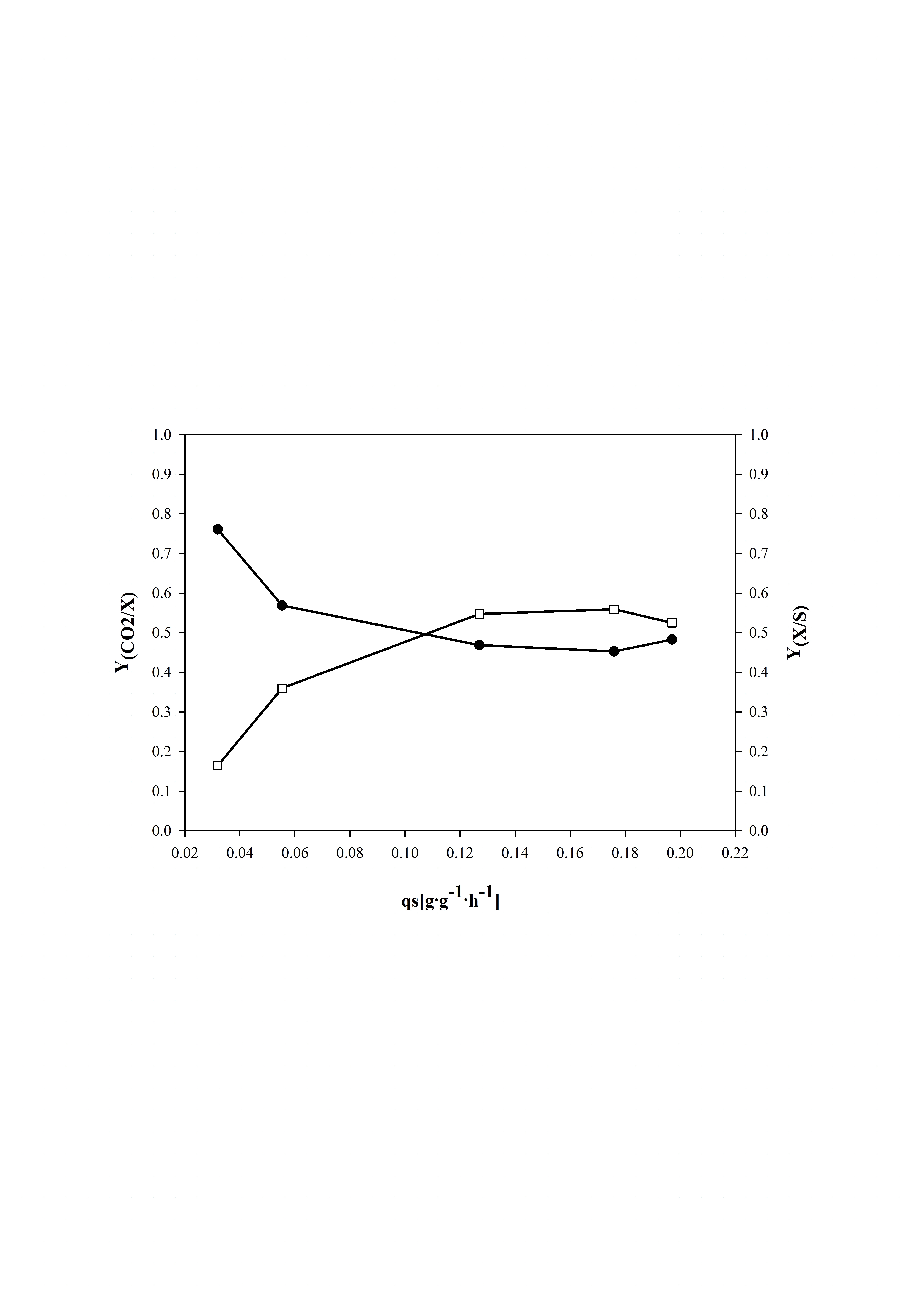

Supplement: Additional file 6: — Figure S6. Carbon dioxide yield (${\text{Y}}_{{{\text{CO}}_{2} /{\text{S}}}}$YCO2/S, black dots) and biomass yield (YX/S, white squares) at different specific sorbitol uptake rates (qs sorbitol). [file 12934_2015_292_MOESM6_ESM.jpg]

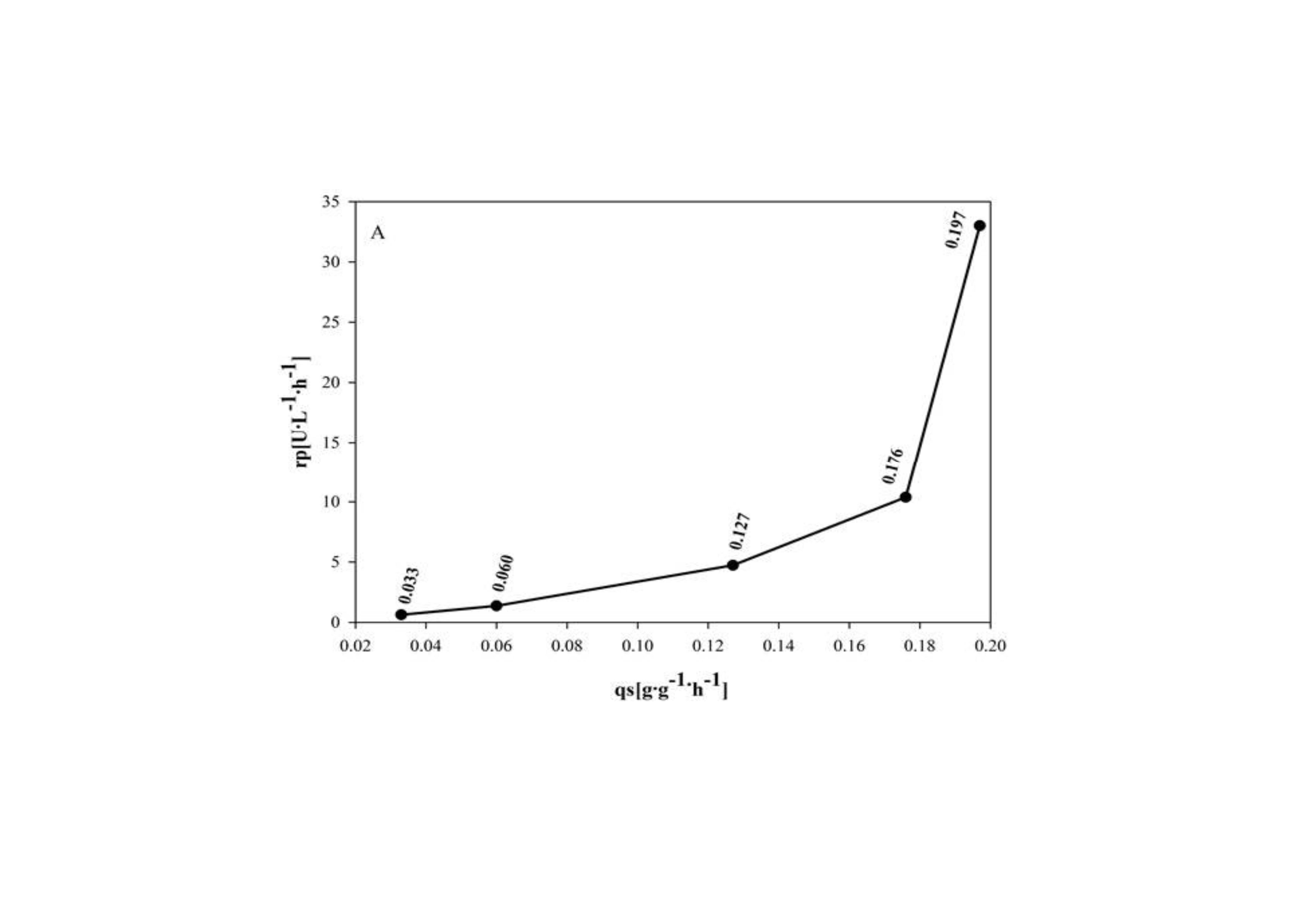

Supplement: Additional file 7: — Figure S7. Volumetric productivity (rp) at different specific sorbitol uptake rates (qs sorbitol). [file 12934_2015_292_MOESM7_ESM.jpg]

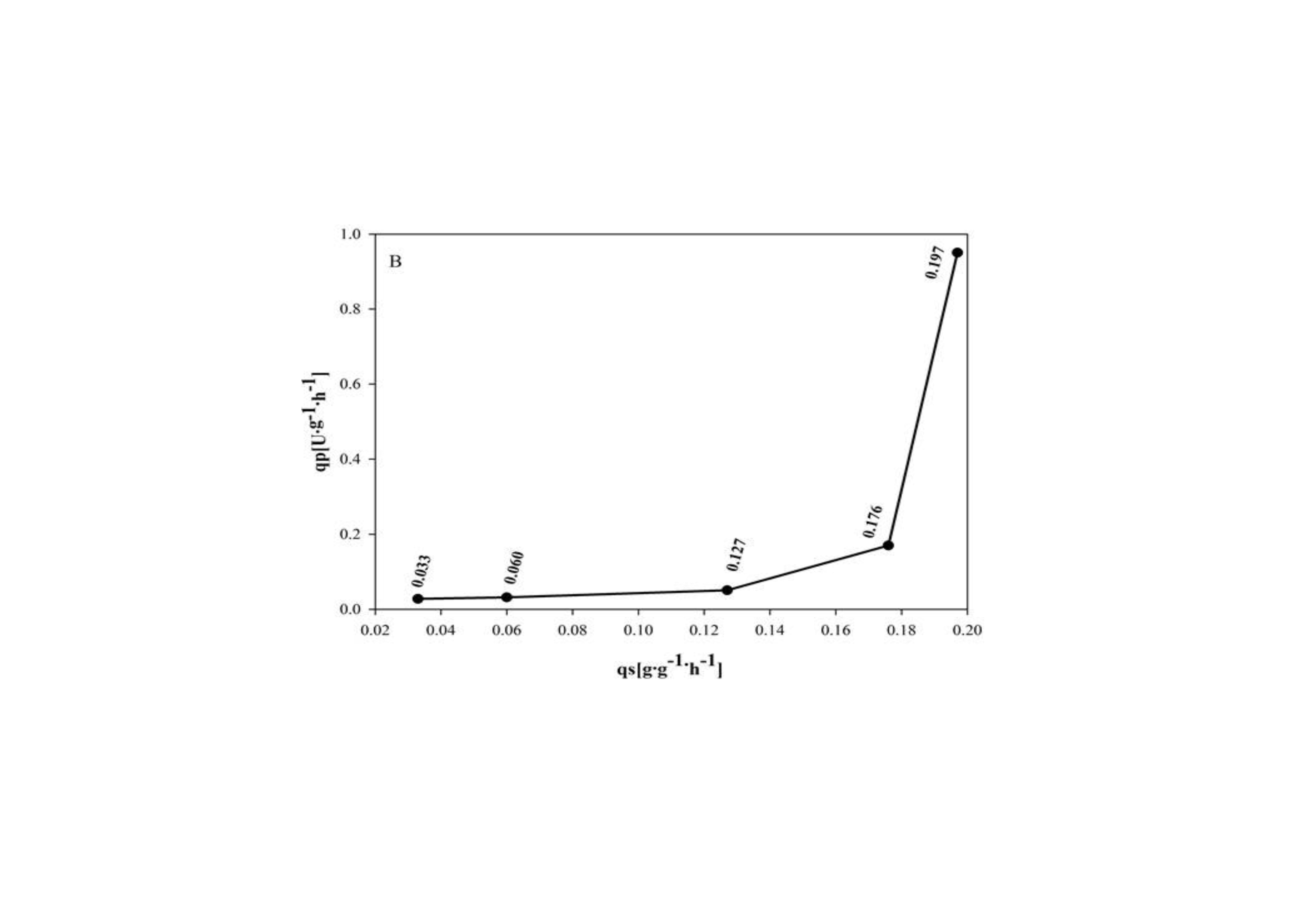

Supplement: Additional file 8: — Figure S8. Specific productivity (qp) at different specific sorbitol uptake rates (qs sorbitol). [file 12934_2015_292_MOESM8_ESM.jpg]
